# A proliferating trichilemmal cyst in the perianal region: A case report

**DOI:** 10.1016/j.ijscr.2018.09.049

**Published:** 2018-10-18

**Authors:** Denise Graffitti D’Avila, Danilo Toshio Kanno, Daniel de Castilho da Silva, Vitor Rafael Pastro, Paula Cristina Stefen Novelli, Bruna Zini de Paula Freitas, Carlos Augusto Real Martinez

**Affiliations:** a University Hospital São Francisco de Assis na Providência de Deus, Bragança Paulista, São Paulo, Brazil; b UNICAMP, Campinas, São Paulo, Brazil

**Keywords:** PTCs, Proliferating trichilemmal cysts, Trichilemmal cyst, Case report, Differential diagnosis, Anal surgery, Epidermoid carcinoma

## Abstract

**Introduction:**

Proliferating trichilemmal cysts (PTCs) are rare benign neoplasms originating from the follicular isthmus. They can undergo trichilemmal keratinization and malignant degeneration to form epidermoid carcinoma. They occur on the scalp in more than 90% of patients and are more common in elderly women.

**Presentation of case:**

A 56-year-old woman complained of perianal discomfort upon sitting. She presented with a perianal nodule of approximately 3 cm in diameter that had exhibited slow and progressive growth over 8 years. After surgical excision of the lesion with safety margins, histopathological examination showed that it was a proliferating trichilemmal cyst in the perianal region.

**Discussion:**

There are no reports in the literature on PTC in the perianal region. The location with the highest prevalence is the scalp; other sites of occurrence are the neck, trunk, underarms, pubis, vulva, lower and upper limbs, upper lip, and gluteal region.

**Conclusion:**

The development of PTC in the perianal region is an exceptional occurrence, and when it does occur, surgical treatment should always be indicated because of the risk of malignant degeneration to form epidermoid carcinoma.

## Highlights

• Proliferating trichilemmal cyst is a rare benign neoplasms from the hair follicles.
• There are no reports of proliferating trichilemmal cyst in the perianal region.
• The treatment for trichilemmal cyst is surgical excision with normal tissue margins.
• The differential diagnosis of trichilemmal cyst include squamous cell carcinoma.

## 1 Introduction

A proliferating trichilemmal cyst is a rare and benign neoplasm originating in the cutaneous annexes and in particular, in the hair follicles. It was first described by Jones in 1966, who gave it the name of proliferating trichilemmal cyst and described it as occurring on or close to the scalp [1,14]. Since then, only over 100 cases have been reported in the literature, but there have been no reported cases of the cyst occurring in the perianal region.

The suggested treatment is surgical excision of the lesion with normal tissue margins. Some reports describe the use of radiotherapy to treat lesions in which malignant degeneration has occurred [2,8].

This case report has been reported in line with the SCARE criteria, surgical case report guidelines [15].

## 2 Presentation of case

A 56-year-old woman sought specialized care, complaining of progressive growth of a nodular lesion on the anus. She reported no pain, bleeding, or changes in intestinal habits and reported slight perianal discomfort upon sitting. She denied previous orificial surgery, and had no history of health problems.

**Fig. 1:**
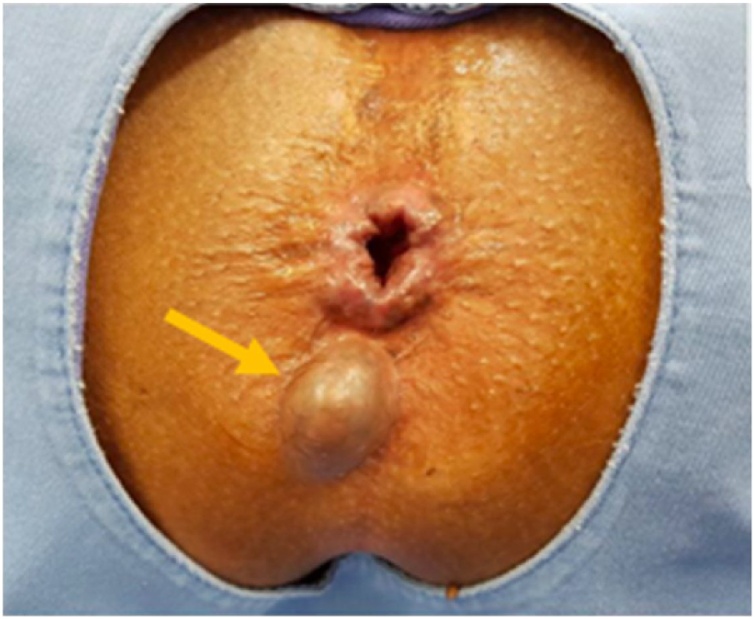
Nodular cystic lesion in the right posterolateral region of the anus.

Proctological examination revealed a nodular cystic lesion in the right posterolateral region of the anus, 2 cm from the mucocutaneous transition zone and measuring 3 cm at its widest diameter. It was covered by a normal epidermis, with no ulcerations or signs of bleeding (Fig. 1).

Upon palpation, the lesion was tender and mobile, with fibroelastic consistency. Upon palpitation of the rectum, there was no bulging, area of fibrosis, or infiltration of the anal canal or rectum wall, and the impression of the sphincter region upon rectal touch was normotonic.

**Fig. 2:**
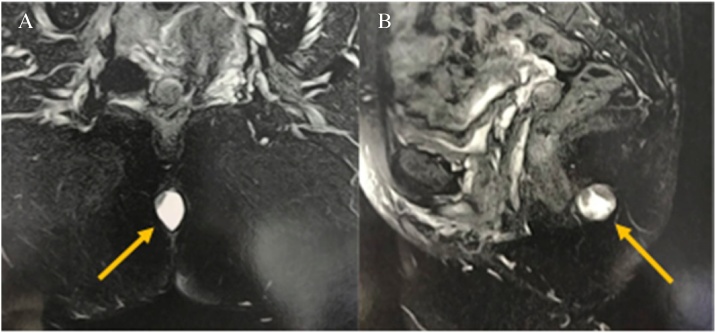
(A, B) Magnetic resonance imaging of the pélvis with a single cystic, nodular image.

Magnetic resonance imaging of the pelvis confirmed the presence of a single cystic, nodular image, described as an ovaloid with mucinous content inside it, located near the anal margin in the posterior median line, with regular contours and well-defined limits. The examination also showed that the lesion measured 2.5 × 1.7 × 2.2 cm, was not invading the sphincter muscle and rectal wall, and did not involve the coccyx or regional lymph node (Fig. 2A, B).

**Fig. 3:**
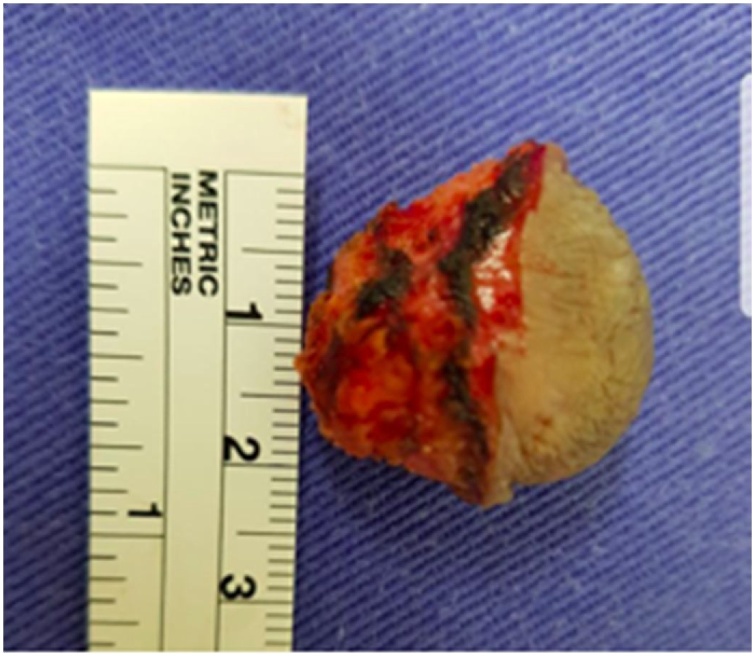
Nodular cystic excision.

The proposed treatment was surgical resection of the lesion. The patient was referred to the surgical department. She was administered spinal anesthesia in the lithotomy position to excise the nodule; 1 cm circumferential safety margins were preserved (Fig. 3). Primary closure of the surgical wound was performed. When the excised piece was dissected, its cystic nature was confirmed, and it was found to contain a brownish mucus.

**Fig. 4:**
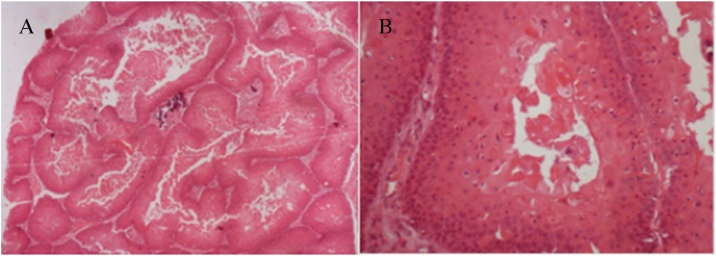
(A, B) Histology demonstrating squamous lesion with trichilemmal keratinization and largely comprised squamous cells with abrupt keratinization and containing hyaline areas.

**Fig. 5:**
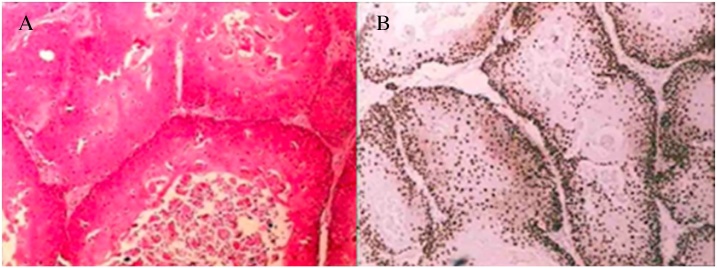
(A, B) Ki-67 demonstrated low mitotic index and CD34 expression, to differentiate the PTC from squamous cell carcinoma, also confirmed the diagnosis of trichilemmal cysts.

Histopathological examination of the excised specimen revealed a squamous lesion with trichilemmal keratinization and largely comprised squamous cells with abrupt keratinization and containing hyaline areas (Fig. 4A, B). These characteristics resulted in a diagnosis of PTC, which was subsequently confirmed by an immunohistochemistry panel; Ki-67 demonstrated low mitotic index, as well as low expression of p53 and p63, suggesting the lesion was benign. CD34 expression, to differentiate the PTC from squamous cell carcinoma, also confirmed the diagnosis (Fig. 5A, B).

## 3 Discussion

PTC, a benign neoplasm arising from the external epithelial sheath of the hair follicle, is histologically characterized by trichilemmal keratinization, which corresponds to the abrupt transition from nucleated epithelial cells to anucleated keratinized cells, without the formation of a granular layer [3,4,11].

PTC is a rare lesion, as we found in a review of the literature; a search of the PubMed and Lilacs databases resulted in only 187 records, more than 90% of which were cases located on the scalp, particularly in skin exposed to the sun [4]. None of the records reported the occurrence of perianal lesions, and we found only one French case report of a PTC in the ischiorectal fossa [5].

The etiopathogenesis of PTC is still unknown, but in most cases, it appears to develop on the wall of a previous hair follicle cyst as a result of trauma or inflammation. There is a suspected causal relationship with infection by the human papilloma virus, but this is not yet well established [6].

The differential diagnosis should include proliferating trichilemmal tumors and squamous cell carcinoma. Immunohistochemical study is a useful tool in the determination for malignancy. Study of the mitotic count using the Ki-67 index is one option because a high cell proliferation rate is associated with malignancy. Moreover, changes in glycoprotein of accession CD34 and mutations in the p53 suppressor gene may also be related, as well as progression of the lesion to squamous cell carcinoma [7,9,10].

Histologically, the biopsy of the tumor in this patient presented both squamous cell proliferation with abrupt keratinization and abundant eosinophilic cytoplasm, which formed homogenous, dense keratin that filled the cystic space. Areas of epidermoid keratinization were seen, forming corneal pearls. The absence of infiltration of the adjacent stroma allowed it to be differentiated from spinocellular carcinoma [2,8,10,12].

The treatment of choice for PTC is surgical excision with normal tissue margins. There are reports of local invasion in more aggressive tumors, recurrence after resection, and metastasis in the malignant form. Other therapeutic options have been proposed for such cases, including Mohs micrographic surgery, neoadjuvant therapy with radiation therapy, or even the use of the latter as the sole treatment [2,6,10,13].

## 4 Conclusion

PTCs are rare benign lesions; their behavior may be indolent or it may be present in more aggressive forms as malignant proliferating trichilemmal tumors. This report of a case of PTC in an uncommon location reminds us to consider this follicular neoplasm in the differential diagnoses, including lesions such as squamous cell carcinoma.

## Conflicts of interest statement

There is no conflicts of interest to declare.

## Funding

There is no sponsors involved.

## Ethical approval

The ethical approval has been given for the ethics committee of the University of São Francisco de Assis de Bragança Paulista, São Paulo, Brazil. Control number 108772/2017.

## Consent

Written informed consent was obtained from the patient for publication of this case report and accompanying images. A copy of the written consent is available for review by the Editor-in-Chief of this journal on request.

## Author contribution

Denise Graffitti D’Avila — Contributions to conception and design.

Danilo Toshio Kanno — Contributions revising it critically for important intellectual content.

Daniel de Castilho da Silva — Revising it critically for important intellectual content.

Vitor Rafael Pastro — Contributions to acquisition of data.

Paula Cristina Stefen Novelli — Contributions to acquisition of data.

Bruna Zini de Paula Freitas — Contributions to analysis and interpretation of data.

Carlos Augusto Real Martinez — Give final approval of the version to be submitted and any revised version.

## Registration of research studies

researchregistry3281.

## Guarantor

Denise Graffitti D’Avila.

## Provenance and peer review

Not commissioned, externally peer-reviewed.
